# One-Shot *GW* Transport Calculations: A Charge-Conserving Solution

**DOI:** 10.1021/acs.jpclett.2c03362

**Published:** 2023-01-20

**Authors:** Dan Klein, Karen Michaeli

**Affiliations:** Department of Condensed Matter Physics, Weizmann Institute of Science, Rehovot 76100, Israel

## Abstract

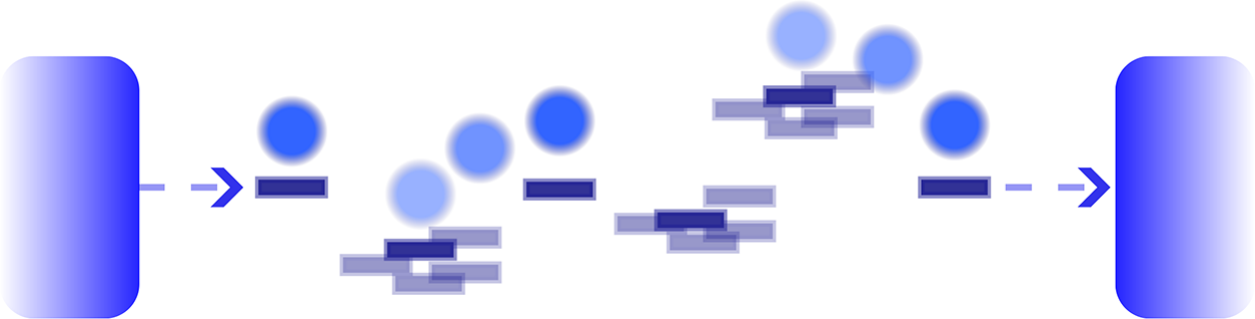

Transport measurements are a common method of characterizing small systems in chemistry and physics. When interactions are negligible, the current through submicrometer structures can be obtained using the Landauer formula. Meir and Wingreen derived an exact expression for the current in the presence of interactions. This powerful tool requires knowledge of the exact Green’s function. Alternatively, self-consistent approximations for the Green’s function are frequently sufficient for calculating the current while crucially satisfying all conservation laws. We provide here yet another alternative, circumventing the high computational cost of these methods. We present expressions for the electric and thermal currents in which the lowest-order self-energy is summed to all orders (one-shot *GW* approximation). We account for both self-energy and vertex corrections such that current is conserved. Our formulas for the currents capture important features due to interactions and, hence, provide a powerful tool for cases in which the exact solution cannot be found.

Transport experiments are arguably the most common method for probing the electronic properties of nanoscopic or microscopic systems. Over the years, such measurements have revealed various fascinating phenomena unique to the submicrometer world. Examples include conductance steps in clean systems,^1,2^ universal conductance fluctuations,^3^ and the Coulomb blockade.^4,5^ Extensive theoretical studies accompanied the experimental effort, and the Landauer formula^6−8^ played a central role in understanding many of the observations. Landauer’s expression describes the current flowing through a finite system connected to leads: electron baths with a well-defined temperature and chemical potential. Specifically, the electric^6^ and thermal^9^ currents are formulated in terms of the scattering probabilities through the finite region. Explaining the observation of conductance steps in ballistic nanowires^1,2^ and predicting the signature of Aharonov Bohm oscillations in small metallic rings^10,11^ are among the many applications of this powerful method. By contrast, the Landauer formula cannot capture the physics behind the appearance of Coulomb blockade oscillations,^12^ long-range electron transfer in molecular chains,^13^ and various other effects that stem from interactions.^14^

Meir and Wingreen^15^ have generalized Landauer’s expression to interacting systems. The many-body Green’s function (GF) replaces the scattering probability in their formulation. The Meir–Wingreen formula (MWF) has successfully explained the transport properties of quantum dots^16,17^ and molecular chains with a few sites.^18^ The original derivation of the MWF assumes knowledge of the exact many-body Green’s function. Later works showed that this condition can be relaxed, and certain approximations to the Green’s function can be applied without violating charge conservation. For example, a self-energy Σ that can be written as the functional derivative of a functional Φ, i.e.
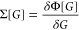
1is guaranteed to be conserving,^19^*provided that the calculation is performed fully self-consistently*.^20^ In cases in which the fully self-consistent approximation for the self-energy is available, the MWF can be further simplified.^17^ Although such approximations can reduce the computational cost, they are still applicable only in systems of limited size.

The *GW* approximation is an example of a Φ-derivable self-energy^21−23^ in which the self-energy Σ = *GW* is a product of the electronic Green’s function *G* and the screened interaction *W*. The latter is calculated self-consistently in the random phase approximation (RPA). Such an approach is typically used to study the effect of electron–electron interactions in molecules and solids.^24−32^ In many cases, its “one-shot” version, *G*_0_*W*_0_, where the self-consistency procedure is forfeited, is sufficient to predict the spectrum of solids^24,25,27−29,31^ very accurately. The interaction *W* can also be replaced by the dressed propagator of the lattice vibrations. Then, the *G*_0_*W*_0_ approximation captures qualitative behavior and scaling with the temperature of many phenomena induced by electron–phonon coupling.^33,34^*G*_0_*W*_0_ is a method used as a compromise between the computationally expensive self-consistent *GW* approximation and the simple, straightforward perturbation expansion in the interaction. The former is typically performed iteratively until convergence is achieved. In each step, the self-energy Σ_(*n*)_ = *G*_(*n*–1)_*W*_(*n*–1)_ is computed using the result of the previous iteration and used to find the GF . Here, *G*_0_ is the non-interacting GF, and similar to *G*_(*n*)_ and Σ_(*n*)_, it is a square matrix proportional to the size of the system. By contrast, the latter does not involve any matrix inversion as *G* = *G*_0_ + *G*_0_Σ_(1)_*G*_0_ for the lowest-order corrections. The *G*_0_*W*_0_ approximation amounts to performing only the first iteration in the *GW* method and finding *G* = *G*_1_.

The successes of the *G*_0_*W*_0_ approximation along with its low computational cost make it an attractive approximation compared to its fully self-consistent counterpart. On the other hand, the literature has noted that in transport calculations, the *G*_0_*W*_0_ scheme is insufficient to uphold current conservation, leading to unphysical predictions. For example, when applied to the Anderson impurity model, it predicts strongly negative differential conductance.^35^ Consequently, the current in large systems is commonly calculated using a straightforward perturbative expansion in the interaction.^36−40^ In these cases, charge conservation is maintained order by order. Going beyond perturbation theory remains a challenge in these systems.^41−43^

In this work, we derive closed formulas for the electric and thermal currents within the *G*_0_*W*_0_ approximation where charge conservation is restored. The strength of our formulas is in their low computational cost; our expression for the currents is a function of the non-interacting electronic Green’s function *G*_0_ and the screened interaction or phonon propagator *W*_0_. Thus, we provide a “one-shot” scheme for calculating the current in large systems such as long molecules, without resorting to straightforward perturbation theory. Specifically, our expressions for the currents contain all contributions with the lowest-order correction to the electronic self-energy (see Figure 1).

**Figure 1:**
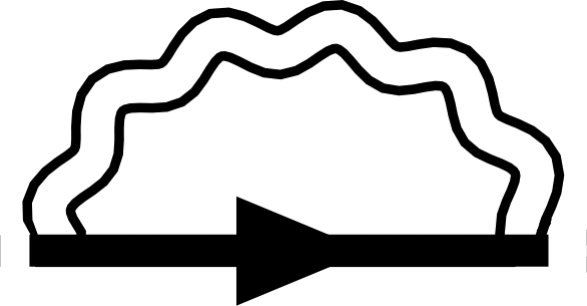
Illustration of the lowest-order approximation of the self-energy given by eq 8a. The self-energy consists of one bare (non-interacting) electronic GF (single line) and one propagator of the boson mode (double wavy line). The latter is given by eq 9 for interactions with large baths of bosons and by eq S8 for electron–electron interactions or small baths.

An essential feature of our formulas is that we include all self-energy and vertex corrections at the same level of approximation, thus conserving charge. The main part of the paper focuses on electron–phonon interactions as a representative example of coupling between the electronic system and any bosonic modes. The modifications required for including electron–electron interactions are given in the Supporting Information. To demonstrate the strength of our results, we calculate the electric current of several model interacting systems using our formula. (i) We study phonon-induced charge transfer through a disordered molecular bridge. Within a straightforward perturbation expansion in the interaction, a non-zero current first arises at orders comparable to the lattice size. By contrast, our expression instantly captures the formation of a polaronic band. (ii) We derive the current through a quantum dot coupled to vibrational modes. We show that using the naïve *G*_0_*W*_0_ approximation in the MWF does not capture the formation of multiple resonance peaks observed in the self-consistent *GW* method.^17^ Moreover, it results in a strong violation of current conservation. Our modified expression for the current restores this symmetry while describing also higher-order features. (iii) Finally, we find that the lack of current conservation resulting from simple insertion of *G*_0_*W*_0_ into the MWF can also lead to violation of Onsager–Casimir relations.^45,46^ As an example, we study the current through a three-dot ring structure pierced by an Aharonov–Bohm flux and subject to electron–phonon interactions. We demonstrate that our formula, by contrast, upholds both current conservation and the Onsager–Casimir relation while increasing the computational load only slightly.

Following ref (15), we study a finite system of interacting electrons connected to two infinite, non-interacting leads
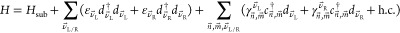
2where  creates an electron with state *m⃗* on site *n⃗* inside the finite subsystem. The index *m⃗* contains all quantum numbers characterizing the electronic state such as the spin and orbital. Similarly,  creates an electron in a state  in the left (right) lead. Both leads are at equilibrium with temperature *T*_L/R_ and chemical potential μ_L/R_. Such a description applies to systems of any dimension. The information on the leads’ positions is encoded in the coupling parameter . The Hamiltonian *H*_sub_ describes the electrons inside the finite subsystem and their interactions with each other or the environment. The latter contains all modes not coupled directly to the leads, for instance, phonons.

The electric current flowing from the subsystem to the *j*th lead (*j* = L/R) is given by the MWF
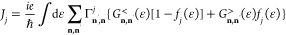
3where *G*_**n**′,**n**_ is the fully dressed Keldysh GF^48^ and < and > denote its lesser and greater components, respectively. For the sake of simplicity, we combine all indices into a single one, , and hence, the Keldysh GF can be written in terms of the single-electron operators as . The GF entering the MWF is a function of the frequency, i.e., the Fourier transform of *G*_**n**,**n**′_(*t*). The bare (non-interacting) current vertex is , and *f*_*j*_(ε) is the Fermi–Dirac distribution function of the same lead. The parameter  is the density of states in lead *j* per quantum number ν⃗, while  transforms the basis of states in the lead to that of the subsystem. The coupling to the leads and the interactions both renormalize the lesser GF
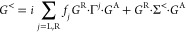
4Here and below, we use dots to denote the product of matrices, i.e., . We replace the lesser index with the greater one and the distribution function by −1 + *f*_*j*_ to obtain *G*^>^(ε). The retarded and advanced components of the GF are
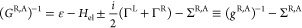
5The Hamiltonian *H*_el_ describes the electrons of the finite subsystem alone in the absence of interactions. We define *g* to be the electron GF in the subsystem, including only the effects of the leads, while the self-energy Σ(ε) is fully dressed with interactions. We include only the lowest-order corrections to the self-energy Σ^(1)^, i.e., apply the GW approximation.

Substitution of the self-energy in eqs 3–5 with the approximate one, Σ → Σ^(1)^, generically results in a spurious violation of current conservation. This difficulty becomes apparent in systems with sufficiently complex dynamics and has therefore been mostly overlooked. By contrast, the exact Landauer formula and the MWF clearly satisfy the equation *J*_L_ = −*J*_R_.^47^ Thus, such a simple substitution is not a consistent approximation of the current. We note that the self-energy consists of a correction to the single-particle spectrum and a relaxation rate. The latter is a manifestation of electronic transitions between different states by scattering events. Vertex corrections account for the current carried by the scattered electrons, thereby maintaining charge conservation. Following Büttiker’s idea,^49^ the effect of interactions is often studied by attaching additional probes to the system and calculating the current from the Landauer formula. The extra leads introduce scattering rates into the electronic Green’s functions. Charge conservation is guaranteed when the chemical potentials of the Büttiker probes are chosen so that there is no net current flowing from them to the system. Fine-tuning the chemical potentials has the same role as correctly including the self-energy and vertex contributions. Self-consistent calculations of the chemical potentials are straightforward in linear response but become increasingly challenging far from equilibrium.

The discussion presented above illustrates the delicate balance between self-energy and vertex corrections. Furthermore, we see that the condition *J*_L_ + *J*_R_ = 0 can help detect inconsistent approximations of the current. In the Green’s function formalism, charge conservation is encoded in the Dyson equation, which we express in a more revealing form as
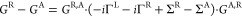
6Up to here, our derivation applies for any subsystem. Next, we use electron–phonon coupling as a prototype for interactions between electrons and any bosonic mode. It can be applied, for example, for electrons in a molecular junction where vibrations play an important role in charge transfer or a quantum dot coupled to an optical cavity. The Hamiltonian of the finite subsystem (*H*_sub_ = *H*_el_ + *H*_ph_ + *H*_el-ph_) is a sum of the non-interacting electron and phonon contributions as well as the coupling between them^50^
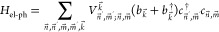
7where  creates a phonon excitation with frequency  and the interaction vertex is generically nondiagonal in space or *m⃗* indices. At the lowest order in the interaction, the components of the self-energy are
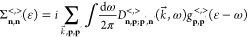
8a
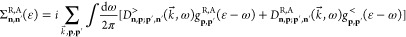
8b

We assume a sizable bosonic bath and therefore neglect the renormalization of the phonon modes. Thus, the phonon propagator maintains a simple form
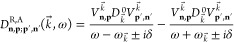
9The lesser and greater components are *D*^<^(ω) = *N*^ph^(ω)[*D*^R^(ω) – *D*^A^(ω)] and *D*^>^(ω) = [1 + *N*^ph^(ω)][*D*^R^(ω) – *D*^A^(ω)], respectively; *N*^ph^(ω) is the Bose–Einstein distribution with phonon temperature *T*_ph_. In the Supporting Information, we include corrections to the bosonic modes within the RPA and derive the current in the presence of electron–electron interactions.

We first consider equal temperatures *T*_L/R_ = *T*_ph_ and derive the electric current flowing from the interacting subsystem to the left lead as a response to an applied voltage *V* = (μ_L_ – μ_R_)/*e*. The current is written using its diagrammatic representation (Figure 2)
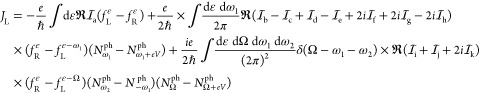
10where  refers to the product of electron and phonon GFs as indicated by diagram α. The voltage enters the equation through the Fermi–Dirac distribution functions of the two leads {}. The diagrams include the bare (single line) and dressed (double line) electronic GFs. Notice that the phonon propagators explicitly shown in the diagrams (single wavy lines) are different from those entering the self-energy (double wavy line). The former denotes the combination , while the latter (eq 9) also includes the real part. The full expressions for each diagram are given in the Supporting Information. The current flowing from the subsystem to the right lead *J*_R_ is obtained by interchanging the left and right leads everywhere in eq 10. Crucially, the currents satisfy the equation *J*_L_ + *J*_R_ = 0 (see the Supporting Information).

**Figure 2:**
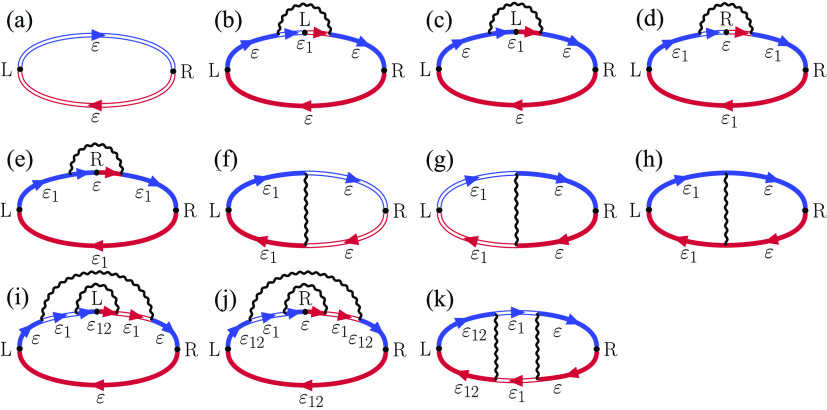
Diagrammatic representation of contributions to the currents given by eqs 10 and 14. The single and double lines are the bare and dressed electronic Green’s function, respectively (see eq 5). We use the color code to indicate the retarded (blue) and advanced (red) GFs. The indices L and R denote the leads from which the GFs end or start, and ε_1_ = ε – ω_1_ while ε_12_ = ε – ω_1_ – ω_2_. The single wavy line represents the combination *D*^R^ – *D*^A^, in contrast to the double line entering the self-energy (Figure 1).

We now apply our expression for the current on three different examples of electronic systems coupled to phonons. We begin with a study of transport through a disordered wire assisted by optical phonons
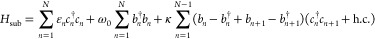
11The on-site energies in the first term are randomly drawn from a uniform distribution in the domain ε_*n*_ ∈ [−*W*, *W*]. This model describes charge transfer through a molecular bridge^13,51^ or a one-dimensional system in the small polaron limit.^52,53^ Importantly, no current can flow in the absence of interactions. Within perturbation theory in the interaction, we must expand to an order equal to or higher than the lattice length (*N* = 5 in our example) to obtain a non-zero current. High order is needed because emission or absorption of phonons accompanies each hopping event. The current, computed according to our method, is shown in Figure 3 for the relationships *W* = ω_0_, Γ^L/R^ = κ = *T*_ph_ = 0.25ω_0_, and μ_L_ = −3.75ω_0_. Our expression given by eq 10 consists of corrections to all orders in Σ^(1)^. Consequently, it captures the formation of a conduction band, evident from the sigmoidal curve, when straightforwardly applied to the model Hamiltonian in eq 11. This simple example already illustrates the power of our derivation.

**Figure 3:**
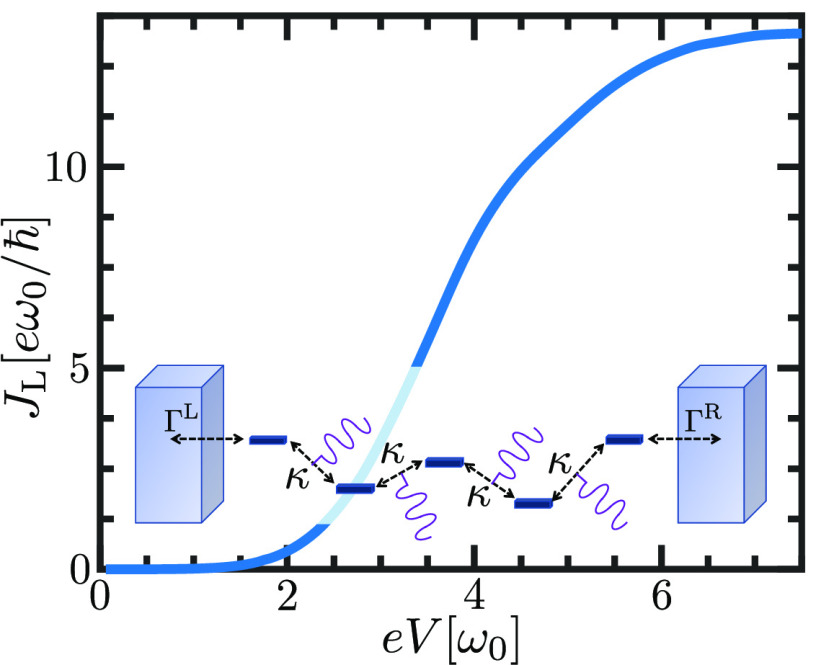
Phonon-assisted current–voltage characteristic. The current as a function of *eV* = μ_L_ – μ_R_ is calculated using eq 10 for the Hamiltonian in eq 11. This Hamiltonian is a toy model of molecular chains or systems in the small polaron limit. Our expression for the current captures the formation of an electronic band of a non-zero width as a consequence of electron–phonon interactions.

Our second example is the current through a single-level quantum dot coupled to a phonon bath. An exact solution for the GF in this system is impossible to find, and thus, the MWF cannot be straightforwardly applied. By contrast, the MWF can be used with a GF and self-energy that are solved within the self-consistent *GW* approximation.^17^ We emphasize that our approximation for the current is not meant to replace such calculations where they are within reach. Rather, we offer an alternative method where they are inapplicable. Nevertheless, this example nicely demonstrates the advantages of our formula over the simple insertion of the *G*_0_*W*_0_ self-energy into the MWF. In both approaches, a single iteration of the *GW* approximation is performed to find the GF. In our method, this GF is inserted into the 10 different contributions in eq 10. The naïve MWF derivation amounts to calculating the sum of  and . Consequently, the calculation time of the two approaches is comparable.

The model Hamiltonian is
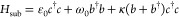
12and to find the current through the system as a function of voltage, we set the chemical potential on the left lead to zero, μ_L_ = 0 and μ_R_ = μ_L_ + *eV*. In Figure 4, we show the first derivative of the current on- and off-resonance. Note that our approximation is valid as long as the approximate self-energy is smaller than the bare GF, and higher-order corrections to the self-energy can be neglected. Thus, we had to consider a lower electron–phonon coupling constant and a stronger coupling to the leads than in ref (17). All other parameters match those in ref (17), and our results agree qualitatively with those reported there. For example, we obtain the strong renormalization of the central peak in the differential conductance in the on-resonance example (Figure 4) or the new peak appearing near *eV* = 0.5*t*_0_ (where *t*_0_ is an arbitrary parameter with units of energy that is used as a scale for all other parameters). By contrast, we find additional peaks in the conductance corresponding to processes involving multiphonon emission and absorption. We demonstrate in Figure 4 that the simple insertion of the *G*_0_*W*_0_ GF into the MWF not only produces dips in the conduction instead of peaks but also leads to violation of current conservation on the order of 10%.

**Figure 4:**
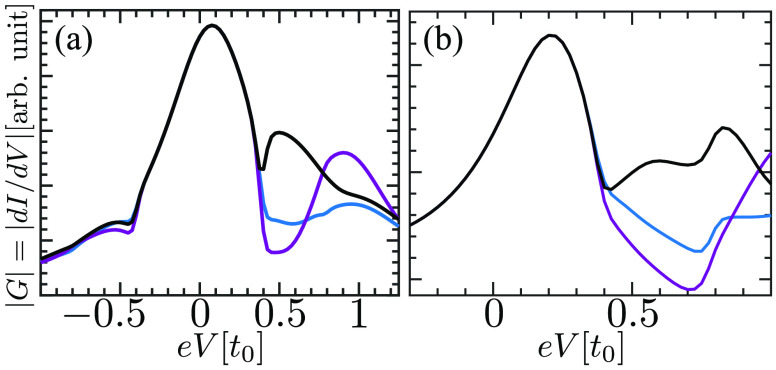
Differential conductance of a single electronic level coupled to a phonon bath. The conductance as a function of voltage is calculated in two methods. The black line is found using eq 10 for the Hamiltonian in eq 12, while the blue and purple lines are found using a simple insertion of *G*_0_*W*_0_ into the MWF, for the left and right leads, respectively. Similar to the derivation in ref (17), the conductance (a) on (ϵ_0_ = 0.3*t*_0_) and (b) off (ϵ_0_ = 0.5*t*_0_) resonance is found at *T* = 0.011*t*_0_, electron–phonon coupling strength γ = 0.26*t*_0_, and phonon frequency ω_0_ = 0.4*t*_0_. By contrast, we use stronger coupling to the lead than in ref (17), Γ^L^ = Γ^R^ = 0.15*t*_0_. Our approximation captures the conductance peaks found by the self-consistent approximation in ref (17). We, however, find additional peaks (for example, near *eV* = 0.8*t*_0_ in the off-resonance case) corresponding to two-phonon absorption.

Our last example focuses on the connection between current conservation and the Onsager–Casimir relations. We show how straightforward substitution of the *G*_0_*W*_0_ approximation in the MWF results in violation of both. On the contrary, our method remedies this spurious ailment. The system is a three-dot Aharonov–Bohm ring subject to electron–phonon interactions, whose Hamiltonian is
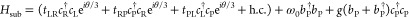
13where the electron–phonon coupling is *g*, *t*_*ij*_ is the hopping amplitude between sites *j* and *i*, and θ is the flux piercing the ring. A similar model was found to exhibit asymmetric thermopower,^44,54^ i.e., Seebeck and Peltier coefficients, α and Π for which Π ≠ *αT*. While such asymmetry is allowed by Onsager–Casimir relations, the two-terminal conductance is constrained to be an even function of the magnetic field. In Figure 5, we show the application of both the naïve *G*_0_*W*_0_ approximation and our method to the system. In the first, the conductance is clearly asymmetric in the flux, while our expression maintains the Onsager–Casimir symmetry.

**Figure 5:**
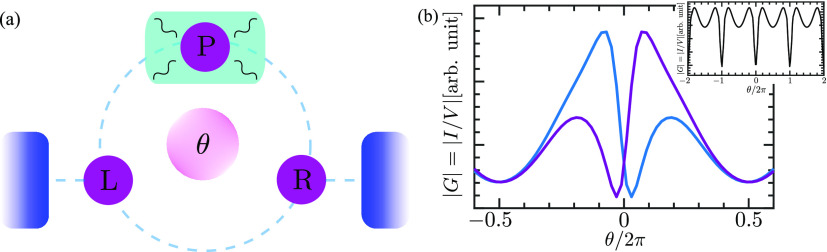
Conductance of the three-site Aharonov–Bohm ring (eq 13) as a function of magnetic flux θ. A schematic representation of the model is shown in panel a. Sites L and R are connected to leads, while site P is subject to electron–phonon interactions. The flux through the system is θ. In panel b, we calculate the conductance once from the current in the left lead (blue) and once from the right lead (purple). Both are the result of a straightforward application of *G*_0_*W*_0_ in the MWF. The two results must coincide if the current is conserved. The lack of current conservation is therefore clearly visible, as well as the violation of Onsager–Casimir relations. By contrast, the current found using our formula given by eq 10, which is shown in the inset, maintains both current conservation and the Onsager–Casimir relation. We set here Γ^L^ = Γ^R^ = 0.25*g*, ω_0_ = 0.5*g*, and the temperature *T* = 0.25*g*.

We conclude with the derivation of the electric and thermal currents induced by both an applied voltage and a temperature difference between the two leads. We find several new processes affecting the thermoelectric and thermal responses that are absent from eq 10. The diagrammatic representations of these additional contributions are shown in Figure 6. Together with the terms in Figure 6, the currents can be written as
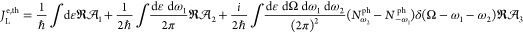
14with
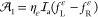
15a
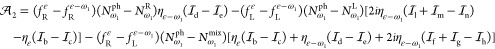
15b
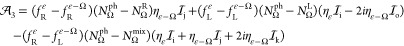
15c

**Figure 6:**
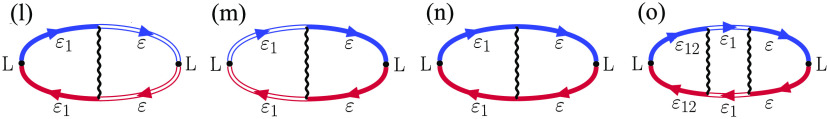
Diagrammatic representation of contributions appearing in the general thermoelectric response. These diagrams, in addition to those given in Figure 2, enter the expression for the electric and heat currents given by eq 14.

The parameter η_ε_ equals −*e* for the electric current and ε – μ_L_ for the thermal current. The temperature entering the Fermi–Dirac distribution function of the left (right) lead is always *T*_L_ (*T*_R_). The Bose–Einstein distribution functions *N*^*j*^ can, on the contrary, depend on the temperature of either the leads *j* = L/R or the phonons *N*^ph^. In addition, we use the shorthand notation , which arises from the identity .

The electric and thermal conductances of free electrons are connected through the Wiedemann–Franz law, which stems from the common origin of the two quantities. Each electron flowing between the two electrodes carries its charge and free energy. The equivalence between the currents and the Wiedemann–Franz law breaks in the presence of interactions.^55−58^ Specifically, an electron that is backscattered after absorbing (emitting) phonons increases (reduces) the thermal current from the subsystem to the lead of its origin. By contrast, the energy in which electrons enter the lead is irrelevant for the electric current. Therefore, the diagrams in Figure 6, accounting for such processes, do not contribute to the electric conductance. Importantly, the electric current in response to a temperature difference between the leads *is* sensitive to backscattering by phonons. The driving force of the current in this case is proportional to the electron’s energy, which changes after the scattering event. Another dissimilarity between the currents is in charge conservation. The total electric current flowing out of the left lead must reach the right one, i.e., *J*_L_ + *J*_R_ = 0, while heat is also transferred to the phonon baths.

In summary, we obtained an approximation for the electric and heat currents in a two-terminal setup through an interacting finite region. This work aimed to find a tractable expression for the currents in cases in which the exact GF or a fully self-consistent approximation of the GF cannot be found, while straightforward perturbation theory does not capture the physical picture accurately. Specifically, we started from the general MWF.^15^ We derived expressions for the currents that sum over the infinite subseries of contributions containing the lowest-order corrections to the self-energy. Such a simplification is known as the *G*_0_*W*_0_ approximation, which is widely used in studies of equilibrium properties of molecules and solids. Our work provides a way of extending *G*_0_*W*_0_ calculations to obtain transport coefficients. In particular, we expect it to be applicable to systems such as molecular junctions, nanotubes, and nanoribbons.

So far, transport calculations through mesoscopic systems were mainly performed within perturbation theory in the interaction. For smaller systems, self-consistent solutions, like the *GW* approximation, are typically applied. Our approach bridges the gap between these two optional methods. As we demonstrated with the examples given above, it can capture more complex interaction processes in comparison to the perturbative approach. Computationally, the cost of our formulas is between those of the two alternatives. The self-consistent calculation consists of several iterations until convergence is achieved. In each iteration, the GF is found through matrix inversion, an operation with the complexity of *O*(*n*^α^), where α ≳ 2.4 and *n* is the system’s size. The matrix inversion is the bottleneck of the self-consistent derivations and can be applied only for sufficiently small structures. By contrast, the GF within perturbation theory does not involve such costly operations and can be performed on large systems. Our approach requires a single matrix inversion operation for the derivation of the GF within the *G*_0_*W*_0_ approximation. In all methods, after the GF and self-energy are obtained, they are inserted into the expression for the current, which consists of several matrix products, an operation with an efficiency similar to that of inversion. Here, our expression includes 10 different terms in comparison to two (MWF) and three (first-order perturbation theory).

Finally, we note that the formulas derived in this paper are based on the non-interacting GF for the electronic subsystem. In many applications of the GW approximation, however, the initial GF is obtained using other methods, such as self-consistent Hartree–Fock (for example, see ref (35)) or density functional theory.^59^ These techniques, though including the effects of interactions on a mean-field level, are formally equivalent to adding a static potential to the effective Hamiltonian for the subsystem. The corresponding GF in the absence of additional (dynamical) interactions can be used as *G*_0_ in our calculation. This is due to the fact that static potentials do not lead to any vertex corrections to the current.

## Supporting Information Available

Modifications required for electron–electron interactions, corrections to the bosonic modes within the RPA, and the expressions for the diagrams contributing to the current, including proof of current conservation (PDF)
