# Supplementary material for: One-Shot GW Transport Calculations: A Charge-Conserving Solution
Source: J Phys Chem Lett. 2023 Jan 20;14(4):897–904. doi: 10.1021/acs.jpclett.2c03362 (PMC11163472; doi:10.1021/acs.jpclett.2c03362)
Supplement: Supplementary file 1 — jz2c03362_si_001.pdf [file jz2c03362_si_001.pdf]

One-shot GW Transport Calculations - a Charge Conserving Solution - Supporting Information

Dan Klein and Karen Michaeli
Department of Condensed Matter Physics, Weizmann Institute of Science, Rehovot 76100, Israel

EXPLICIT EXPRESSION FOR THE CURRENT

The main results of our paper, Eqs. (10) and (14), are written in terms of the diagrams shown in Fig. 2. For completeness, we give here the explicit expression for each of the diagrams:

$$\Re\mathcal{I}_a = \left[\Gamma^L\cdot G^R\cdot\Gamma^R\cdot G^A\right]^{\varepsilon}_{\mathbf{n},\mathbf{n}}\,; \tag{S1a}$$

$$\Re\mathcal{I}_b = \left[g^R\cdot\Gamma^R\cdot g^A\cdot\Gamma^L\cdot g^R - g^A\cdot\Gamma^L\cdot g^R\cdot\Gamma^R\cdot g^A\right]^{\varepsilon}_{\mathbf{n}',\mathbf{n}}\left[D^R(\omega_1)-D^A(\omega_1)\right]_{\mathbf{n},\mathbf{p};\mathbf{p}',\mathbf{n}'}\left[G^R\cdot\Gamma^L\cdot G^A\right]^{\varepsilon-\omega_1}_{\mathbf{p},\mathbf{p}'}\,; \tag{S1b}$$

$$\Re\mathcal{I}_c = \left[g^R\cdot\Gamma^R\cdot g^A\cdot\Gamma^L\cdot g^R - g^A\cdot\Gamma^L\cdot g^R\cdot\Gamma^R\cdot g^A\right]^{\varepsilon}_{\mathbf{n}',\mathbf{n}}\left[D^R(\omega_1)-D^A(\omega_1)\right]_{\mathbf{n},\mathbf{p};\mathbf{p}',\mathbf{n}'}\left[g^R\cdot\Gamma^L\cdot g^A\right]^{\varepsilon-\omega_1}_{\mathbf{p},\mathbf{p}'}\,; \tag{S1c}$$

$$\Re\mathcal{I}_d = \left[g^R\cdot\Gamma^R\cdot g^A\cdot\Gamma^L\cdot g^R - g^A\cdot\Gamma^L\cdot g^R\cdot\Gamma^R\cdot g^A\right]^{\varepsilon-\omega_1}_{\mathbf{n}',\mathbf{n}}\left[D^R(\omega_1)-D^A(\omega_1)\right]_{\mathbf{p}',\mathbf{n}';\mathbf{n},\mathbf{p}}\left[G^R\cdot\Gamma^R\cdot G^A\right]^{\varepsilon}_{\mathbf{p},\mathbf{p}'}\,; \tag{S1d}$$

$$\Re\mathcal{I}_e = \left[g^R\cdot\Gamma^R\cdot g^A\cdot\Gamma^L\cdot g^R - g^A\cdot\Gamma^L\cdot g^R\cdot\Gamma^R\cdot g^A\right]^{\varepsilon-\omega_1}_{\mathbf{n}',\mathbf{n}}\left[D^R(\omega_1)-D^A(\omega_1)\right]_{\mathbf{p}',\mathbf{n}';\mathbf{n},\mathbf{p}}\left[g^R\cdot\Gamma^R\cdot g^A\right]^{\varepsilon}_{\mathbf{p},\mathbf{p}'}\,; \tag{S1e}$$

$$\Re\mathcal{I}_f = \left[g^A\cdot\Gamma^L\cdot g^R\right]^{\varepsilon-\omega_1}_{\mathbf{n}',\mathbf{n}}\left[D^R(\omega_1)-D^A(\omega_1)\right]_{\mathbf{p}',\mathbf{n}';\mathbf{n},\mathbf{p}}\left[G^R\cdot\Gamma^R\cdot G^A\right]^{\varepsilon}_{\mathbf{p},\mathbf{p}'}\,; \tag{S1f}$$

$$\Re\mathcal{I}_g = \left[G^A\cdot\Gamma^L\cdot G^R\right]^{\varepsilon-\omega_1}_{\mathbf{n}',\mathbf{n}}\left[D^R(\omega_1)-D^A(\omega_1)\right]_{\mathbf{p}',\mathbf{n}';\mathbf{n},\mathbf{p}}\left[g^R\cdot\Gamma^R\cdot g^A\right]^{\varepsilon}_{\mathbf{p},\mathbf{p}'}\,; \tag{S1g}$$

$$\Re\mathcal{I}_h = \left[g^A\cdot\Gamma^L\cdot g^R\right]^{\varepsilon-\omega_1}_{\mathbf{n}',\mathbf{n}}\left[D^R(\omega_1)-D^A(\omega_1)\right]_{\mathbf{p}',\mathbf{n}';\mathbf{n},\mathbf{p}}\left[g^R\cdot\Gamma^R\cdot g^A\right]^{\varepsilon}_{\mathbf{p},\mathbf{p}'}\,; \tag{S1h}$$

$$\begin{aligned}\Re\mathcal{I}_i = &\left[g^R\cdot\Gamma^R\cdot g^A\cdot\Gamma^L\cdot g^R - g^A\cdot\Gamma^L\cdot g^R\cdot\Gamma^R\cdot g^A\right]^{\varepsilon}_{\mathbf{n}',\mathbf{n}}\left[D^R(\omega_1)-D^A(\omega_1)\right]_{\mathbf{n},\mathbf{p};\mathbf{p}',\mathbf{n}'}\\ &\times G^R_{\mathbf{p},\mathbf{m}}(\varepsilon-\omega_1)\left[D^R(\omega_2)-D^A(\omega_2)\right]_{\mathbf{m},\mathbf{q};\mathbf{q}',\mathbf{m}'}\left[g^R\cdot\Gamma^L\cdot g^A\right]^{\varepsilon-\omega_1-\omega_2}_{\mathbf{q},\mathbf{q}'}G^A_{\mathbf{m}',\mathbf{p}'}(\varepsilon-\omega_1);\end{aligned} \tag{S1i}$$

$$\begin{aligned}\Re\mathcal{I}_j = &\left[g^R\cdot\Gamma^R\cdot g^A\cdot\Gamma^L\cdot g^R - g^A\cdot\Gamma^L\cdot g^R\cdot\Gamma^R\cdot g^A\right]^{\varepsilon-\omega_1-\omega_2}_{\mathbf{n}',\mathbf{n}}\left[D^R(\omega_2)-D^A(\omega_2)\right]_{\mathbf{p}',\mathbf{n}';\mathbf{n},\mathbf{p}}\\ &\times G^R_{\mathbf{p},\mathbf{m}}(\varepsilon-\omega_1)\left[D^R(\omega_1)-D^A(\omega_1)\right]_{\mathbf{q}',\mathbf{m}';\mathbf{m},\mathbf{q}}\left[g^R\cdot\Gamma^R\cdot g^A\right]^{\varepsilon}_{\mathbf{q},\mathbf{q}'}G^A_{\mathbf{m}',\mathbf{p}'}(\varepsilon-\omega_1);\end{aligned} \tag{S1j}$$

$$\begin{aligned}\Re\mathcal{I}_k = &\left[g^A\cdot\Gamma^L\cdot g^R\right]^{\varepsilon-\omega_1-\omega_2}_{\mathbf{n}',\mathbf{n}}\left[D^R(\omega_2)-D^A(\omega_2)\right]_{\mathbf{p}',\mathbf{n}';\mathbf{n},\mathbf{p}}\\ &\times G^R_{\mathbf{p},\mathbf{m}}(\varepsilon-\omega_1)\left[D^R(\omega_1)-D^A(\omega_1)\right]_{\mathbf{q}',\mathbf{m}';\mathbf{m},\mathbf{q}}\left[g^R\cdot\Gamma^R\cdot g^A\right]^{\varepsilon}_{\mathbf{q},\mathbf{q}'}G^A_{\mathbf{m}',\mathbf{p}'}(\varepsilon-\omega_1);\end{aligned} \tag{S1k}$$

In all of the above equations, we sum over repeated indices and denote the product of matrices by a dot, $[A\cdot B]^{\varepsilon}_{\mathbf{n},\mathbf{n}'} = A_{\mathbf{n},\mathbf{p}}(\varepsilon)B_{\mathbf{p},\mathbf{n}'}(\varepsilon) = \sum_{\mathbf{p}} A_{\mathbf{n},\mathbf{p}}(\varepsilon)B_{\mathbf{p},\mathbf{n}'}(\varepsilon)$.

Similarly, we can write the expressions for the additional diagrams in Fig. 6

$$\Re\mathcal{I}_l = \left[g^A\cdot\Gamma^L\cdot g^R\right]^{\varepsilon-\omega_1}_{\mathbf{n}',\mathbf{n}} \left[D^R(\omega_1) - D^A(\omega_1)\right]_{\mathbf{p}',\mathbf{n}';\mathbf{n},\mathbf{p}} \left[G^R\cdot\Gamma^L\cdot G^A\right]^{\varepsilon}_{\mathbf{p},\mathbf{p}'}; \tag{S2a}$$

$$\Re\mathcal{I}_m = \left[G^A\cdot\Gamma^L\cdot G^R\right]^{\varepsilon-\omega_1}_{\mathbf{n}',\mathbf{n}} \left[D^R(\omega_1) - D^A(\omega_1)\right]_{\mathbf{p}',\mathbf{n}';\mathbf{n},\mathbf{p}} \left[g^R\cdot\Gamma^L\cdot g^A\right]^{\varepsilon}_{\mathbf{p},\mathbf{p}'}; \tag{S2b}$$

$$\Re\mathcal{I}_n = \left[g^A\cdot\Gamma^L\cdot g^R\right]^{\varepsilon-\omega_1}_{\mathbf{n}',\mathbf{n}} \left[D^R(\omega_1) - D^A(\omega_1)\right]_{\mathbf{p}',\mathbf{n}';\mathbf{n},\mathbf{p}} \left[g^R\cdot\Gamma^L\cdot g^A\right]^{\varepsilon}_{\mathbf{p},\mathbf{p}'}; \tag{S2c}$$

$$\begin{aligned}\Re\mathcal{I}_o = {} & \left[g^A\cdot\Gamma^L\cdot g^R\right]^{\varepsilon-\omega_1-\omega_2}_{\mathbf{n}',\mathbf{n}} \left[D^R(\omega_2) - D^A(\omega_2)\right]_{\mathbf{n},\mathbf{p};\mathbf{p}',\mathbf{n}'} \\ & \times G^R_{\mathbf{p},\mathbf{m}}(\varepsilon-\omega_1)\left[D^R(\omega_1) - D^A(\omega_1)\right]_{\mathbf{q}',\mathbf{m}';\mathbf{m},\mathbf{q}} \left[g^R\cdot\Gamma^L\cdot g^A\right]^{\varepsilon}_{\mathbf{m},\mathbf{m}'} G^A_{\mathbf{m}',\mathbf{p}'}(\varepsilon-\omega_1);\end{aligned} \tag{S2d}$$

CURRENT CONSERVATION

This section aims to show that our expression for the electric current does not violate charge conservation. Below, we show that currents flowing between the subsystem and the left and right leads satisfy $J_{\mathrm{L}} + J_{\mathrm{R}} = 0$. To generate the expression for J_{R}, we replace the left and right leads in Eq. (10). In preparation to add the two currents, we shift the fermion's frequency under the integral in J_{R} as follows $\varepsilon \to \varepsilon + \omega$ (or $\varepsilon \to \varepsilon + \omega_1 + \omega_2$). Similarly, we substitute $\omega_i \to -\omega_i$ for all bosons' frequencies. Next, we write the sum of the two currents as

$$J_{\mathrm{L}} + J_{\mathrm{R}} = J^{(0)} + J^{(1)} + J^{(2)}. \tag{S3}$$

Here, $J^{(i)}$ is the contribution to the currents from diagrams in which i phononic propagators explicitly appear. Namely, $J^{(0)}$ contains $\mathcal{I}_a$ from Fig. 2 of the main text and its equivalent contribution to the current in the right lead. The term $J^{(1)}$ contains diagrams $\mathcal{I}_b$-$\mathcal{I}_h$, and the remaining diagrams enter $J^{(2)}$. Next, we simplify the expressions for $J^{(1)}$ and $J^{(2)}$ by applying the identity given by Eq. (6) in the main text on the bare Green's function. In addition, we use the relation $N^{\mathrm{ph}}_{\omega_1}N^{\mathrm{ph}}_{\omega_2} = N^{\mathrm{ph}}_{\omega_1+\omega_2}\left[N^{\mathrm{ph}}_{\omega_1} - N^{\mathrm{ph}}_{-\omega_2}\right]$. Thus, the different contributions to $J_{\mathrm{L}} + J_{\mathrm{R}}$ can be written as

$$J^{(0)} = -\frac{e}{\hbar}\int \mathrm{d}\varepsilon\,[f^{\varepsilon}_{\mathrm{L}} - f^{\varepsilon}_{\mathrm{R}}]\left[\Gamma^L\cdot G^R\cdot\Gamma^R\cdot G^A - \Gamma^R\cdot G^R\cdot\Gamma^L\cdot G^A\right]^{\varepsilon}_{\mathbf{n},\mathbf{n}}; \tag{S4a}$$

$$\begin{aligned}J^{(1)} = {} & i\frac{e}{\hbar}\int\frac{\mathrm{d}\varepsilon\mathrm{d}\omega_1}{2\pi}\left[f^{\varepsilon}_{\mathrm{R}} - f^{\varepsilon-\omega_1}_{\mathrm{L}}\right]\left[N^{\mathrm{ph}}_{\omega_1} - N^{\mathrm{ph}}_{\omega_1+eV}\right]\left[D^R(\omega_1) - D^A(\omega_1)\right]_{\mathbf{n},\mathbf{p};\mathbf{p}',\mathbf{n}'} \\ & \left\{\left[g^R\cdot\Gamma^L\cdot g^A\right]^{\varepsilon-\omega_1}_{\mathbf{p},\mathbf{p}'}\left[G^R\cdot\Gamma^R\cdot G^A - G^A\cdot\Gamma^R\cdot G^R\right]^{\varepsilon}_{\mathbf{n}',\mathbf{n}} - \left[g^R\cdot\Gamma^R\cdot g^A\right]^{\varepsilon}_{\mathbf{n}',\mathbf{n}}\left[G^R\cdot\Gamma^L\cdot G^A - G^A\cdot\Gamma^L\cdot G^R\right]^{\varepsilon-\omega_1}_{\mathbf{p},\mathbf{p}'}\right\};\end{aligned} \tag{S4b}$$

$$\begin{aligned}J^{(2)} = {} & \frac{e}{\hbar}\int\frac{\mathrm{d}\varepsilon\mathrm{d}\omega_1\mathrm{d}\omega_2}{(2\pi)^2}\left[f^{\varepsilon}_{\mathrm{R}} - f^{\varepsilon-\omega_1-\omega_2}_{\mathrm{L}}\right]\left[N^{\mathrm{ph}}_{\omega_2} - N^{\mathrm{ph}}_{-\omega_1}\right]\left[N^{\mathrm{ph}}_{\omega_1+\omega_2} - N^{\mathrm{ph}}_{\omega_1+\omega_2+eV}\right] \\ & \times\left[D^R(\omega_1) - D^A(\omega_1)\right]_{\mathbf{n},\mathbf{p};\mathbf{p}',\mathbf{n}'}\left[D^R(\omega_2) - D^A(\omega_2)\right]_{\mathbf{m},\mathbf{q};\mathbf{q}',\mathbf{m}'}\left[g^R\cdot\Gamma^R\cdot g^A\right]^{\varepsilon}_{\mathbf{n}',\mathbf{n}} \\ & \times\left[G^R_{\mathbf{p},\mathbf{m}}(\varepsilon-\omega_1)G^A_{\mathbf{m}',\mathbf{p}'}(\varepsilon-\omega_1) - G^A_{\mathbf{p},\mathbf{m}}(\varepsilon-\omega_1)G^R_{\mathbf{m}',\mathbf{p}'}(\varepsilon-\omega_1)\right]\left[g^R\cdot\Gamma^L\cdot g^A\right]^{\varepsilon-\omega_1-\omega_2}_{\mathbf{q},\mathbf{q}'}.\end{aligned} \tag{S4c}$$

Although each term seems to have a different number of phonon propagators, it comprises an infinite sum of corrections of all orders in the interaction. This infinite sum is generated by the fully dressed Green's functions G. Consequently, each term is generally not zero, and current conservation is restored only upon summing all terms.

Our next step is to sum $J^{(0)}$ and $J^{(1)}$. For this purpose, we use Eq. (6) in the main text to rewrite $J^{(0)}$ as a function of the self-energy

$$J^{(0)} = -i\frac{e}{2\hbar}\int \mathrm{d}\varepsilon\, [f_{\rm L}^{\varepsilon} - f_{\rm R}^{\varepsilon}] \left[G^A\cdot(\Gamma^R-\Gamma^L)\cdot G^R - G^R\cdot(\Gamma^R-\Gamma^L)\cdot G^A\right]^{\varepsilon}_{\mathbf{n}',\mathbf{n}} \left[\Sigma^R-\Sigma^A\right]^{\varepsilon}_{\mathbf{n},\mathbf{n}'} \tag{S5}$$
$$= i\frac{e}{2\hbar}\int\frac{\mathrm{d}\varepsilon \mathrm{d}\omega_1}{2\pi}\left[f_{\rm R}^{\varepsilon} - f_{\rm L}^{\varepsilon-\omega_1}\right]\left[N^{\rm ph}_{\omega_1} - N^{\rm ph}_{\omega_1+eV}\right]\left[D^R(\omega_1)-D^A(\omega_1)\right]_{\mathbf{n},\mathbf{p};\mathbf{p}',\mathbf{n}'}\left\{\left[g^R\cdot\Gamma^R\cdot g^A\right]^{\varepsilon}_{\mathbf{n}',\mathbf{n}}\left[G^A\cdot(\Gamma^R-\Gamma^L)\cdot G^R\right.\right.$$
$$\left.\left.-G^R\cdot(\Gamma^R-\Gamma^L)\cdot G^A\right]^{\varepsilon-\omega_1}_{\mathbf{p},\mathbf{p}'} + \left[G^A\cdot(\Gamma^R-\Gamma^L)\cdot G^R - G^R\cdot(\Gamma^R-\Gamma^L)\cdot G^A\right]^{\varepsilon}_{\mathbf{n}',\mathbf{n}}\left[g^R\cdot\Gamma^L\cdot g^A\right]^{\varepsilon-\omega_1}_{\mathbf{p},\mathbf{p}'}\right\}.$$

We, thus, brought $J^{(0)}$ into a form similar to $J^{(1)}$ and their sum yields

$$J^{(0)}+J^{(1)} = i\frac{e}{2\hbar}\int\frac{\mathrm{d}\varepsilon \mathrm{d}\omega_1}{2\pi}\left[f_{\rm R}^{\varepsilon} - f_{\rm L}^{\varepsilon-\omega_1}\right]\left[N^{\rm ph}_{\omega_1} - N^{\rm ph}_{\omega_1+eV}\right]\left[D^R(\omega_1)-D^A(\omega_1)\right]_{\mathbf{n},\mathbf{p};\mathbf{p}',\mathbf{n}'} \tag{S6}$$
$$\left\{\left[g^R\cdot\Gamma^R\cdot g^A\right]^{\varepsilon}_{\mathbf{n}',\mathbf{n}}\left[G^A\cdot(\Gamma^R+\Gamma^L)\cdot G^R - G^R\cdot(\Gamma^R+\Gamma^L)\cdot G^A\right]^{\varepsilon-\omega_1}_{\mathbf{p},\mathbf{p}'}\right.$$
$$\left.+\left[G^R\cdot(\Gamma^R+\Gamma^L)\cdot G^A - G^A\cdot(\Gamma^R+\Gamma^L)\cdot G^R\right]^{\varepsilon}_{\mathbf{n}',\mathbf{n}}\left[g^R\cdot\Gamma^L\cdot g^A\right]^{\varepsilon-\omega_1}_{\mathbf{p},\mathbf{p}'}\right\}.$$

Finally, we apply again Eq. (6) to rewrite the above equation

$$J^{(0)}+J^{(1)} = \frac{e}{2\hbar}\int\frac{\mathrm{d}\varepsilon \mathrm{d}\omega_1}{2\pi}\left[f_{\rm R}^{\varepsilon} - f_{\rm L}^{\varepsilon-\omega_1}\right]\left[N^{\rm ph}_{\omega_1} - N^{\rm ph}_{\omega_1+eV}\right]\left[D^R(\omega_1)-D^A(\omega_1)\right]_{\mathbf{n},\mathbf{p};\mathbf{p}',\mathbf{n}'} \tag{S7}$$
$$\times\left\{\left[g^R\cdot\Gamma^R\cdot g^A\right]^{\varepsilon}_{\mathbf{n}',\mathbf{n}}\left[G^A_{\mathbf{p},\mathbf{m}}(\varepsilon-\omega_1)G^R_{\mathbf{m}',\mathbf{p}'}(\varepsilon-\omega_1) - G^R_{\mathbf{p},\mathbf{m}}(\varepsilon-\omega_1)G^A_{\mathbf{m}',\mathbf{p}'}(\varepsilon-\omega_1)\right]\left[\Sigma^R(\varepsilon-\omega_1)-\Sigma^A(\varepsilon-\omega_1)\right]_{\mathbf{m},\mathbf{m}'}\right.$$
$$\left.+\left[G^R_{\mathbf{n}',\mathbf{m}}(\varepsilon)G^A_{\mathbf{m}',\mathbf{n}}(\varepsilon) - G^A_{\mathbf{n}',\mathbf{m}}(\varepsilon)G^R_{\mathbf{m}',\mathbf{n}}(\varepsilon)\right]\left[\Sigma^R(\varepsilon)-\Sigma^A(\varepsilon)\right]_{\mathbf{m},\mathbf{m}'}\left[g^R\cdot\Gamma^L\cdot g^A\right]^{\varepsilon-\omega_1}_{\mathbf{p},\mathbf{p}'}\right\}$$
$$=\frac{e}{\hbar}\int\frac{\mathrm{d}\varepsilon \mathrm{d}\omega_1 \mathrm{d}\omega_2}{(2\pi)^2}\left[g^R\cdot\Gamma^R\cdot g^A\right]^{\varepsilon}_{\mathbf{n}',\mathbf{n}}\left[G^A_{\mathbf{p},\mathbf{m}}(\varepsilon-\omega_1)G^R_{\mathbf{m}',\mathbf{p}'}(\varepsilon-\omega_1) - G^R_{\mathbf{p},\mathbf{m}}(\varepsilon-\omega_1)G^A_{\mathbf{m}',\mathbf{p}'}(\varepsilon-\omega_1)\right]\left[g^R\cdot\Gamma^L\cdot g^A\right]^{\varepsilon-\omega_1-\omega_2}_{\mathbf{q},\mathbf{q}'}$$
$$\times\left[f_{\rm R}^{\varepsilon} - f_{\rm L}^{\varepsilon-\omega_1-\omega_2}\right]\left[N^{\rm ph}_{\omega_1+\omega_2} - N^{\rm ph}_{\omega_1+\omega_2+eV}\right]\left[N^{\rm ph}_{\omega_2} - N^{\rm ph}_{-\omega_1}\right]\left[D^R(\omega_1)-D^A(\omega_1)\right]_{\mathbf{n},\mathbf{p};\mathbf{p}',\mathbf{n}'}\left[D^R(\omega_2)-D^A(\omega_2)\right]_{\mathbf{m},\mathbf{q};\mathbf{q}',\mathbf{m}'}$$

We found that $J^{(0)}+J^{(1)}=-J^{(2)}$ and, hence, our expression for the current conserves charge.

THE ELECTRIC CURRENT IN THE PRESENCE OF ELECTRON-ELECTRON INTERACTIONS

We devote this section to modifying the expression for the current to include renormalization of the Boson properties by the interactions. Such a scenario is relevant for small bosonic baths and electron-electron interactions, where the renormalization is significant. We start by considering a system coupled to a phonon bath, i.e., we return to the example given in the main text when only a voltage bias is applied to the system. We derived the current for systems where the renormalization of the phonons properties is negligible. Consequently, the Boson propagator used in the main text is simply $D^{R/A}(\vec{k},\omega)\propto[(\omega-\omega_{\vec{k}}\pm i\delta)^{-1}-(\omega+\omega_{\vec{k}}\pm i\delta)^{-1}]$. By contrast, here we are interested in systems for which this expression is no longer valid, and the dressed propagator becomes

$$\left[U^{R,A}\right]^{-1} = \left[D^{R,A}\right]^{-1} - \Pi^{R/A}. \tag{S8}$$

The lesser and greater components of the Green's function are

$$U^{<,>}(\vec{k},\omega) = U^R\cdot\Pi^{<,>}\cdot U^A. \tag{S9}$$

To proceed with the derivation of the current, we need to specify the form of the phonons self-energy (polarization operator) Π. Here, we derived the phonons' self-energy within the random phase approximation (RPA)

$$\Pi^{R,A}_{\mathbf{n},\mathbf{p};\mathbf{p}',\mathbf{n}'}(\omega) = -\sum_{j=L/R}\int\frac{\mathrm{d}\varepsilon}{2\pi}\left\{f_j(\varepsilon)\left[g^R\Gamma^j g^A\right]^{\varepsilon}_{\mathbf{n}',\mathbf{n}} g^{A,R}_{\mathbf{p},\mathbf{p}'}(\varepsilon-\omega) + f_j(\varepsilon-\omega)g^{R,A}_{\mathbf{n}',\mathbf{n}}(\varepsilon)\left[g^R\Gamma^j g^A\right]^{\varepsilon-\omega}_{\mathbf{p},\mathbf{p}'}\right\}. \tag{S10}$$

We use the expression for the retard and advanced components of the self-energy to introduce a new quantity

$$\Pi^R_{\mathbf{n},\mathbf{p};\mathbf{p}',\mathbf{n}'}(\omega) - \Pi^A_{\mathbf{n},\mathbf{p};\mathbf{p}',\mathbf{n}'}(\omega) = -i \sum_{j,\ell=L/R} \int \frac{d\varepsilon}{2\pi} \left[f_j(\varepsilon) - f_\ell(\varepsilon-\omega)\right] (g^R\Gamma^j g^A)^{\varepsilon}_{\mathbf{n}',\mathbf{n}} (g^R\Gamma^\ell g^A)^{\varepsilon-\omega}_{\mathbf{p},\mathbf{p}'} \equiv \sum_{j,\ell=R/L} \left[\Pi^R_{j,\ell}(\omega) - \Pi^A_{j,\ell}(\omega)\right]_{\mathbf{n},\mathbf{p};\mathbf{p}',\mathbf{n}'} . \tag{S11}$$

The indices j and ℓ in $\Pi^R_{j,\ell}(\omega) - \Pi^A_{j,\ell}(\omega)$ denote the leads through which the Green's function in the polarization operator pass and relax into. Consequently, the lesser and greater components of Π can be written as

$$\Pi^< = \sum_{j,\ell=R/L} N^{\mathrm{ph}}_{\omega+\mu_\ell-\mu_j} \left[\Pi^R_{j,\ell} - \Pi^A_{j,\ell}\right]; \tag{S12a}$$

$$\Pi^> = \sum_{j,\ell=R/L} \left[1 + N^{\mathrm{ph}}_{\omega+\mu_\ell-\mu_j}\right] \left[\Pi^R_{j,\ell} - \Pi^A_{j,\ell}\right]. \tag{S12b}$$

Finally, similar to Eq. (S11), we define $U^R_{j,\ell}(\omega) - U^A_{j,\ell}(\omega)$

$$U^R - U^A = \sum_{j,\ell=L/R} \left[U^R \cdot (\Pi^R_{j,\ell} - \Pi^A_{j,\ell}) \cdot U^A\right] \equiv \sum_{j,\ell=L/R} \left[U^R_{j,\ell} - U^A_{j,\ell}\right]. \tag{S13}$$

We can now write the expression for the current using the diagrammatic representation appearing in the main text, similar to Eq. (10). In cases where the renormalization of the boson modes is significant, the double wiggly line entering the self-energy (Fig. 1) represents the full phonon propagator (S8) and (S9). The single wiggly line in Figs. 2 and 6 denotes $U^R_{j,\ell} - U^A_{j,\ell}$. We demonstrate the changes in $\mathcal{I}_\alpha$ on $\mathcal{I}_b$ (Eq. (S1b)) that becomes

$$\Re\mathcal{I}_b = \sum_{j,\ell=L/R} \left[g^R\cdot\Gamma^R\cdot g^A\cdot\Gamma^L\cdot g^R - g^A\cdot\Gamma^L\cdot g^R\cdot\Gamma^R\cdot g^A\right]^{\varepsilon}_{\mathbf{n}',\mathbf{n}} \left[U^R_{j,\ell}(\omega_1) - U^A_{j,\ell}(\omega_1)\right]_{\mathbf{n},\mathbf{p};\mathbf{p}',\mathbf{n}'} \left[G^R\cdot\Gamma^L\cdot G^A\right]^{\varepsilon-\omega_1}_{\mathbf{p},\mathbf{p}'} . \tag{S14}$$

Then, we can write the expression for the current with the modified $\mathcal{I}_\alpha$

$$\begin{aligned} J_{\mathrm{L}} = &-\frac{e}{\hbar}\int d\varepsilon \Re\mathcal{I}_a \left[f^\varepsilon_{\mathrm{L}} - f^\varepsilon_{\mathrm{R}}\right] \\ &+ \frac{e}{2\hbar}\int \frac{d\varepsilon d\omega_1}{2\pi} \sum_{j_1,\ell_1=L/R} \Re\left[\mathcal{I}_b - \mathcal{I}_c + \mathcal{I}_d - \mathcal{I}_e + 2i\mathcal{I}_f + \mathcal{I}_g - \mathcal{I}_h\right] \left[f^\varepsilon_{\mathrm{R}} - f^{\varepsilon-\omega_1}_{\mathrm{L}}\right] \left[N^{\mathrm{ph}}_{\omega_1+\Delta\mu_{j_1,\ell_1}} - N^{\mathrm{ph}}_{\omega_1+eV}\right] \\ &+ \frac{e}{2\hbar}\int \frac{d\varepsilon d\omega_1}{2\pi} \sum_{j_1,\ell_1=L/R} \left\{\Re\left[\mathcal{I}_b - \mathcal{I}_c - 2i\mathcal{I}_l + \mathcal{I}_m - \mathcal{I}_n\right]\left[f^\varepsilon_{\mathrm{L}} - f^{\varepsilon-\omega_1}_{\mathrm{L}}\right] + \Re\left[\mathcal{I}_d - \mathcal{I}_e\right]\left[f^\varepsilon_{\mathrm{R}} - f^{\varepsilon-\omega_1}_{\mathrm{R}}\right]\right\} \left[N^{\mathrm{ph}}_{\omega_1} - N^{\mathrm{ph}}_{\omega_1+\Delta\mu_{j_1,\ell_1}}\right] \\ &+ i\frac{e}{2\hbar}\int \frac{d\varepsilon d\omega_1 d\omega_2}{(2\pi)^2} \sum_{j_1,j_2,\ell_1,\ell_2=L/R} \Re\left[\mathcal{I}_i + \mathcal{I}_j + 2i\mathcal{I}_k\right] \left[f^\varepsilon_{\mathrm{R}} - f^{\varepsilon-\omega_1-\omega_2}_{\mathrm{L}}\right] \left[N^{\mathrm{ph}}_{\omega_1+\omega_2+\Delta\mu_{j_1,\ell_1}+\Delta\mu_{j_2,\ell_2}} - N^{\mathrm{ph}}_{\omega_1+\omega_2+eV}\right] \\ &\times \left[N^{\mathrm{ph}}_{\omega_2+\Delta\mu_{j_2,\ell_2}} - N^{\mathrm{ph}}_{\omega_1+\Delta\mu_{j_1,\ell_1}}\right] \\ &+ i\frac{e}{2\hbar}\int \frac{d\varepsilon d\omega_1 d\omega_2}{(2\pi)^2} \sum_{j_1,j_2,\ell_1,\ell_2=L/R} \left\{\Re\left[\mathcal{I}_i - 2i\mathcal{I}_o\right]\left[f^\varepsilon_{\mathrm{L}} - f^{\varepsilon-\omega_1-\omega_2}_{\mathrm{L}}\right] + \Re\mathcal{I}_j\left[f^\varepsilon_{\mathrm{R}} - f^{\varepsilon-\omega_1-\omega_2}_{\mathrm{R}}\right]\right\} \left[N^{\mathrm{ph}}_{\omega_2+\Delta\mu_{j_2,\ell_2}} - N^{\mathrm{ph}}_{-\omega_1-\Delta\mu_{j_1,\ell_1}}\right] \\ &\times \left[N^{\mathrm{ph}}_{\omega_1+\omega_2} - N^{\mathrm{ph}}_{\omega_1+\omega_2+\Delta\mu_{j_1,\ell_1}+\Delta\mu_{j_2,\ell_2}}\right]. \end{aligned} \tag{S15}$$

Here $\Delta\mu_{j,\ell} = \mu_j - \mu_\ell$. Applying the same procedure as in the previous section, we verified that the current is conserved.

So far, we discussed only coupling to boson baths; we turn now to address electron-electron interactions

$$H_{\mathrm{int}} = \sum_{n,n',\vec{m},\vec{m}',} V_{n',\vec{m}';n,\vec{m}} c^\dagger_{n',\vec{m}'} c_{n',\vec{m}'} c^\dagger_{n,\vec{m}} c_{n,\vec{m}} \tag{S16}$$

Neglecting renormalization effects, the propagator of the field mediating the interaction is $D^{R/A}_{\mathbf{n},\mathbf{p};\mathbf{p}',\mathbf{n}'}(\vec{k},\omega) = V_{\vec{n}';\vec{n}}\delta_{\vec{n},\vec{p}}\delta_{\vec{n}',\vec{p}'}$ and $D^{</>}_{\mathbf{n},\mathbf{p};\mathbf{p}',\mathbf{n}'}(\vec{k},\omega) = 0$. Accounting for self-energy corrections, we find that Eqs. (S10)-(S13) also describe the boson propagator here. Correspondingly, Eq. (S15) is also valid for the current through a system with dominant electron-electron interactions.
